# 4-(2-{2-[2-(2-Nitro-1*H*-imidazol-1-yl)ethoxy]eth­oxy}eth­oxy)benzaldehyde

**DOI:** 10.1107/S1600536811014322

**Published:** 2011-04-22

**Authors:** Shu-Xian Li, Da-Hai Zhang, Hoong-Kun Fun, Madhukar Hemamalini

**Affiliations:** a Department of Chemistry, Handan College, Handan, Hebei Province 056005, People’s Republic of China; b X-ray Crystallography Unit, School of Physics, Universiti Sains Malaysia, 11800 USM, Penang, Malaysia

## Abstract

In the mol­ecule of the title compound, C_16_H_19_N_3_O_6_, the imidazole ring is essentially planar [maximum deviation = 0.002 (2) Å] and forms a dihedral angle of 5.08 (14)° with the nitro group. In the crystal structure, adjacent mol­ecules are connected *via* inter­molecular C—H⋯O hydrogen bonds into columns parallel to the *a* axis.

## Related literature

For details and applications of nitro­imidazole, see: Abdel-Jalil *et al.* (2006 ▶); Kennedy *et al.* (2006 ▶); Nagasawa *et al.* (2006 ▶); Nunn *et al.* (1995 ▶). For bond-length data, see: Allen *et al.* (1987 ▶). For the stability of the temperature controller used in the data collection, see: Cosier & Glazer (1986 ▶).
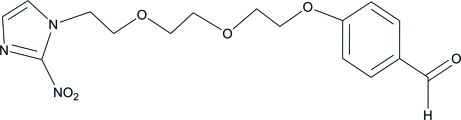


## Experimental

### 

#### Crystal data

C_16_H_19_N_3_O_6_
*M* *_r_* = 349.34
Orthorhombic, 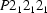

*a* = 4.4403 (3) Å
*b* = 11.4686 (8) Å
*c* = 31.2763 (19) Å
*V* = 1592.72 (18) Å^3^
*Z* = 4
Mo *K*α radiation
μ = 0.11 mm^−1^
*T* = 100 K
1.00 × 0.10 × 0.09 mm

#### Data collection

Bruker SMART APEXII CCD area-detector diffractometer
Absorption correction: multi-scan (*SADABS*; Bruker, 2009 ▶) *T*_min_ = 0.895, *T*_max_ = 0.990
11825 measured reflections
2763 independent reflections
2243 reflections with *I* > 2σ(*I*)
*R*_int_ = 0.045

#### Refinement

*R*[*F* ^2^ > 2σ(*F* ^2^)] = 0.047
*wR*(*F* ^2^) = 0.094
*S* = 1.03
2763 reflections
226 parameters
H-atom parameters constrained
Δρ_max_ = 0.26 e Å^−3^
Δρ_min_ = −0.22 e Å^−3^

### 

Data collection: *APEX2* (Bruker, 2009 ▶); cell refinement: *SAINT* (Bruker, 2009 ▶); data reduction: *SAINT*; program(s) used to solve structure: *SHELXTL* (Sheldrick, 2008 ▶); program(s) used to refine structure: *SHELXTL*; molecular graphics: *SHELXTL*; software used to prepare material for publication: *SHELXTL* and *PLATON* (Spek, 2009 ▶).

## Supplementary Material

Crystal structure: contains datablocks global, I. DOI: 10.1107/S1600536811014322/rz2583sup1.cif

Structure factors: contains datablocks I. DOI: 10.1107/S1600536811014322/rz2583Isup2.hkl

Additional supplementary materials:  crystallographic information; 3D view; checkCIF report

## Figures and Tables

**Table 1:** Hydrogen-bond geometry (Å, °)

*D*—H⋯*A*	*D*—H	H⋯*A*	*D*⋯*A*	*D*—H⋯*A*
C9—H9*B*⋯O4^i^	0.97	2.56	3.335 (3)	137
C10—H10*A*⋯O4^ii^	0.97	2.57	3.461 (3)	152

Symmetry codes: (i) 
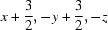
; (ii) 
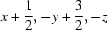
.

**Fig. 1.**
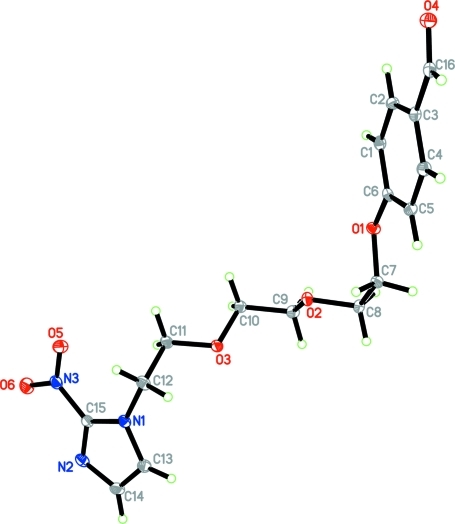
The asymmetric unit of the title compound, showing 30% probability displacement ellipsoids.

**Fig. 2.**
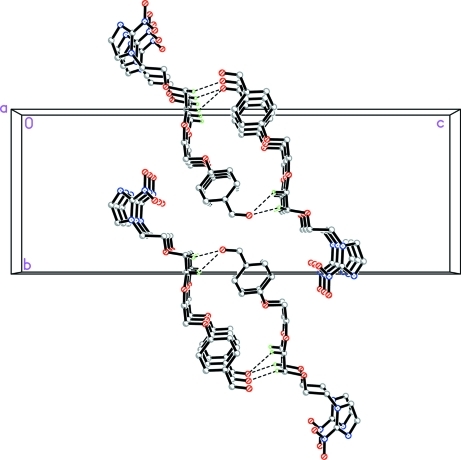
The crystal packing of the title compound viewed along the a axis. H atoms non involved in hydrogen bonds are omitted.

## supplementary crystallographic information

### Comment

Nitroimidazole is an important building block in the design and synthesis of hypoxia makers (Abdel-Jalil *et al.* 2006; Kennedy *et al.* 2006, Nagasawa *et al.*, 2006). In a normal cell, the nitroimidazole moiety undergoes reduction to become a potentially reactive species and can be reoxidized in the presence of normal oxygen levels. However in hypoxic tissues, the low oxygen concentration is not able to effectively reoxidize the molecule and this results in more reactive intermediates that bind with the components of hypoxic tissues (Nunn *et al.*, 1995). In an attempt to develop new hypoxic cell radiosensitizers, we present herein the crystal structure of 4-(2-(2-(2-(2-nitro-1*H*-imidazol-1-yl) ethoxy)-ethoxy)ethoxy)benzaldehyde (I).

In (I), (Fig. 1), the imidazole group is essentially planar, with a maximum deviation of 0.002 (2) Å for atom N2. The nitro group is twisted from the mean plane of imidazole ring with torsion angles O5—N3—C15—N1 = -3.7 (3)° and O6—N3—C15—N1 = 176.7 (2)°. The conformation of the 1-(2-(2-ethoxy)ethoxy)ethyl)propane group is (-)-syn-clinal with respect to the imidazole ring, which is reflected by the torsion angle N1—C12—C11—O3 = -105.5 (2)°. The dihedral angle between the imidazole (N1–N2/C13–C15) ring and the benzene (C1–C6) ring is 38.60 (13)°.  Bond distances and angles have normal values (Allen *et al.*, 1987).

The crystal packing (Fig. 2) shows that the molecules are linked by weak intermolecular C9—H9B···O4 and C10—H10A···O4 (Table 1) hydrogen interactions into columns parallel to the *a* axis.

### Experimental

To a solution of the 4-(2-(2-(2-(2-nitro-1*H*-imidazol-1-yl)ethoxy) ethoxy)ethyl-4-methylbenzenesulfonate (0.600 g, 1.5 mmol) and potassium carbonate (0.569 g, 4.1 mmol) in DMF (20 mL) was added a solution of 4-hydroxybenzaldehyde (0.166 g, 1.4 mmol) in DMF (10 ml) under argon atmosphere. The mixture was stirred at 120°C for 20 h. After concentration on the rotary evaporator under reduced pressure, ethyl acetate (80 ml) was then added to the reaction residue. The content was then washed with water (20 ml × 3), dried (Na_2_SO_4_) and the organic layer was evaporated to dryness and subjected to chromatography on silica with EtOAc–hexane (3:1 *v*/*v*) to afford the desired compound (I) (0.435 g, yield 89%). Analysis Calcd for C_16_H_19_N_3_O_6_: C 55.01, H 5.48, N 12.03%; found: C 55.31, H 4.91, N 12.43%. ^1^H NMR (500 MHz, CDCl_3_) δ: 3.66 (m, 4H), 3.86 (m, 4H), 4.22 (t, J = 4.5 Hz, 2H), 4.64 (t, J = 4.5 Hz, 2H), 7.04 (d, J = 9.0, 2H), 7.10 (s, 1H), 7.23 (s, 1H), 7.87 (d, J = 9.0 Hz, 2H), 9.91 (s, 1H). Single crystals of X-ray diffraction quality were prepared by the slow diffusion of hexane into a dichloromethane solution of the title compound.

### Refinement

All H atoms were positioned geometrically [C—H = 0.93 or 0.97 Å] and were refined using a riding model, with *U*_iso_(H) = 1.2*U*_eq_(C). In the absence of significant anomalous scattering effects, 1735 Friedel pairs were merged.

### Crystal data

**Table:**

C_16_H_19_N_3_O_6_	*F*(000) = 736
*M**_r_* = 349.34	*D*_x_ = 1.457 Mg m^−^^3^
Orthorhombic, *P*2_1_2_1_2_1_	Mo *K*α radiation, λ = 0.71073 Å
Hall symbol: P 2ac 2ab	Cell parameters from 3196 reflections
*a* = 4.4403 (3) Å	θ = 2.6–27.4°
*b* = 11.4686 (8) Å	µ = 0.11 mm^−^^1^
*c* = 31.2763 (19) Å	*T* = 100 K
*V* = 1592.72 (18) Å^3^	Needle, colourless
*Z* = 4	1.00 × 0.10 × 0.09 mm

### Data collection

**Table:**

Bruker SMART APEXII CCD area-detector diffractometer	2763 independent reflections
Radiation source: fine-focus sealed tube	2243 reflections with *I* > 2σ(*I*)
graphite	*R*_int_ = 0.045
φ and ω scans	θ_max_ = 30.2°, θ_min_ = 2.6°
Absorption correction: multi-scan (*SADABS*; Bruker, 2009)	*h* = −6→5
*T*_min_ = 0.895, *T*_max_ = 0.990	*k* = −12→16
11825 measured reflections	*l* = −37→43

### Refinement

**Table:**

Refinement on *F*^2^	Primary atom site location: structure-invariant direct methods
Least-squares matrix: full	Secondary atom site location: difference Fourier map
*R*[*F*^2^ > 2σ(*F*^2^)] = 0.047	Hydrogen site location: inferred from neighbouring sites
*wR*(*F*^2^) = 0.094	H-atom parameters constrained
*S* = 1.03	*w* = 1/[σ^2^(*F*_o_^2^) + (0.0349*P*)^2^ + 0.4321*P*] where *P* = (*F*_o_^2^ + 2*F*_c_^2^)/3
2763 reflections	(Δ/σ)_max_ = 0.001
226 parameters	Δρ_max_ = 0.26 e Å^−^^3^
0 restraints	Δρ_min_ = −0.22 e Å^−^^3^

### Special details

**Table:**

Experimental. The crystal was placed in the cold stream of an Oxford Cryosystems Cobra open-flow nitrogen cryostat (Cosier & Glazer, 1986) operating at 100.0 (1) K.
Geometry. All s.u.'s (except the s.u. in the dihedral angle between two l.s. planes) are estimated using the full covariance matrix. The cell s.u.'s are taken into account individually in the estimation of s.u.'s in distances, angles and torsion angles; correlations between s.u.'s in cell parameters are only used when they are defined by crystal symmetry. An approximate (isotropic) treatment of cell s.u.'s is used for estimating s.u.'s involving l.s. planes.
Refinement. Refinement of F^2^ against ALL reflections. The weighted R-factor wR and goodness of fit S are based on F^2^, conventional R-factors R are based on F, with F set to zero for negative F^2^. The threshold expression of F^2^ > 2σ(F^2^) is used only for calculating R-factors(gt) etc. and is not relevant to the choice of reflections for refinement. R-factors based on F^2^ are statistically about twice as large as those based on F, and R- factors based on ALL data will be even larger.

### Fractional atomic coordinates and isotropic or equivalent isotropic displacement parameters (Å^2^)

**Table:**

	*x*	*y*	*z*	*U*_iso_*/*U*_eq_	
O1	0.9164 (4)	0.80126 (13)	0.06764 (4)	0.0200 (4)	
O2	1.0360 (4)	0.59396 (12)	0.11489 (4)	0.0195 (3)	
O3	0.9292 (4)	0.36579 (13)	0.15473 (4)	0.0219 (4)	
O4	0.0201 (4)	1.14948 (13)	−0.02814 (5)	0.0258 (4)	
O5	0.3451 (4)	0.06021 (15)	0.17441 (5)	0.0295 (4)	
O6	0.5051 (5)	−0.10825 (14)	0.19678 (5)	0.0339 (5)	
N1	0.7409 (5)	0.17301 (15)	0.22943 (5)	0.0195 (4)	
N2	0.8668 (5)	−0.00344 (17)	0.25495 (6)	0.0243 (5)	
N3	0.5073 (5)	−0.00129 (17)	0.19754 (5)	0.0243 (4)	
C1	0.6176 (6)	0.87874 (18)	0.01331 (7)	0.0193 (5)	
H1A	0.6778	0.8169	−0.0039	0.023*	
C2	0.4203 (6)	0.96061 (18)	−0.00235 (7)	0.0193 (5)	
H2A	0.3471	0.9536	−0.0301	0.023*	
C3	0.3288 (5)	1.05450 (19)	0.02312 (7)	0.0190 (5)	
C4	0.4436 (6)	1.06324 (19)	0.06456 (7)	0.0210 (5)	
H4A	0.3840	1.1252	0.0818	0.025*	
C5	0.6446 (6)	0.98164 (19)	0.08066 (7)	0.0201 (5)	
H5A	0.7222	0.9893	0.1081	0.024*	
C6	0.7279 (5)	0.88797 (18)	0.05488 (7)	0.0178 (5)	
C7	1.0208 (6)	0.80108 (18)	0.11112 (6)	0.0202 (5)	
H7A	0.8515	0.7973	0.1307	0.024*	
H7B	1.1339	0.8716	0.1171	0.024*	
C8	1.2175 (6)	0.69626 (19)	0.11638 (7)	0.0207 (5)	
H8A	1.3665	0.6940	0.0937	0.025*	
H8B	1.3226	0.7002	0.1435	0.025*	
C9	1.2191 (6)	0.4924 (2)	0.11083 (7)	0.0214 (5)	
H9A	1.3631	0.4893	0.1341	0.026*	
H9B	1.3299	0.4952	0.0841	0.026*	
C10	1.0220 (6)	0.38626 (18)	0.11178 (6)	0.0202 (5)	
H10A	0.8471	0.3982	0.0937	0.024*	
H10B	1.1322	0.3193	0.1011	0.024*	
C11	0.7292 (6)	0.27044 (19)	0.15794 (7)	0.0224 (5)	
H11A	0.8336	0.1984	0.1514	0.027*	
H11B	0.5644	0.2797	0.1378	0.027*	
C12	0.6082 (7)	0.26698 (19)	0.20368 (7)	0.0243 (6)	
H12A	0.6493	0.3411	0.2174	0.029*	
H12B	0.3914	0.2568	0.2028	0.029*	
C13	0.9408 (6)	0.1895 (2)	0.26212 (7)	0.0242 (5)	
H13A	1.0129	0.2605	0.2721	0.029*	
C14	1.0146 (7)	0.0807 (2)	0.27736 (7)	0.0264 (5)	
H14A	1.1469	0.0666	0.2998	0.032*	
C15	0.7066 (6)	0.05487 (19)	0.22695 (6)	0.0204 (5)	
C16	0.1127 (6)	1.14220 (19)	0.00810 (7)	0.0213 (5)	
H16A	0.0418	1.1961	0.0279	0.026*	

### Atomic displacement parameters (Å^2^)

**Table:**

	*U*^11^	*U*^22^	*U*^33^	*U*^12^	*U*^13^	*U*^23^
O1	0.0253 (9)	0.0186 (7)	0.0161 (7)	0.0025 (8)	0.0000 (7)	−0.0003 (6)
O2	0.0184 (8)	0.0159 (7)	0.0243 (7)	−0.0011 (7)	0.0010 (8)	0.0014 (6)
O3	0.0291 (10)	0.0198 (7)	0.0168 (7)	−0.0051 (8)	−0.0008 (7)	−0.0001 (6)
O4	0.0266 (10)	0.0266 (8)	0.0242 (8)	0.0009 (8)	−0.0027 (8)	0.0016 (6)
O5	0.0294 (10)	0.0345 (9)	0.0245 (8)	−0.0009 (9)	−0.0035 (8)	0.0016 (7)
O6	0.0496 (13)	0.0188 (8)	0.0333 (9)	−0.0068 (9)	0.0004 (11)	−0.0028 (7)
N1	0.0239 (11)	0.0163 (9)	0.0184 (9)	0.0011 (8)	0.0026 (9)	0.0005 (7)
N2	0.0337 (12)	0.0194 (9)	0.0200 (9)	0.0019 (10)	0.0030 (9)	0.0025 (7)
N3	0.0303 (12)	0.0233 (9)	0.0193 (8)	−0.0045 (10)	0.0044 (10)	0.0002 (8)
C1	0.0225 (12)	0.0159 (10)	0.0195 (10)	−0.0018 (10)	0.0037 (10)	−0.0024 (8)
C2	0.0185 (12)	0.0220 (10)	0.0174 (9)	−0.0025 (10)	0.0001 (9)	0.0003 (8)
C3	0.0175 (11)	0.0182 (10)	0.0214 (10)	−0.0024 (10)	0.0023 (10)	0.0005 (8)
C4	0.0235 (12)	0.0179 (10)	0.0216 (10)	0.0008 (11)	0.0004 (10)	−0.0033 (8)
C5	0.0243 (13)	0.0197 (10)	0.0165 (9)	−0.0024 (10)	−0.0008 (10)	−0.0018 (8)
C6	0.0182 (11)	0.0148 (10)	0.0205 (10)	−0.0016 (10)	0.0034 (10)	0.0027 (8)
C7	0.0245 (13)	0.0191 (10)	0.0170 (9)	−0.0026 (11)	0.0000 (11)	0.0004 (8)
C8	0.0198 (12)	0.0200 (10)	0.0222 (10)	−0.0042 (10)	−0.0006 (11)	0.0005 (9)
C9	0.0224 (12)	0.0190 (10)	0.0227 (10)	0.0035 (11)	−0.0003 (11)	−0.0009 (9)
C10	0.0251 (13)	0.0157 (10)	0.0199 (10)	0.0021 (10)	−0.0012 (11)	−0.0002 (8)
C11	0.0300 (14)	0.0151 (10)	0.0220 (11)	−0.0005 (10)	−0.0028 (12)	0.0028 (9)
C12	0.0314 (15)	0.0157 (10)	0.0258 (11)	0.0037 (11)	0.0012 (11)	0.0029 (9)
C13	0.0268 (13)	0.0272 (11)	0.0185 (10)	−0.0019 (12)	0.0027 (11)	−0.0034 (9)
C14	0.0333 (14)	0.0273 (11)	0.0185 (10)	0.0009 (12)	0.0006 (12)	0.0000 (9)
C15	0.0270 (13)	0.0169 (10)	0.0174 (10)	−0.0017 (10)	0.0027 (10)	−0.0010 (8)
C16	0.0193 (12)	0.0172 (10)	0.0274 (11)	−0.0024 (10)	0.0010 (11)	0.0005 (9)

### Geometric parameters (Å, °)

**Table:**

O1—C6	1.360 (3)	C4—H4A	0.9300
O1—C7	1.437 (2)	C5—C6	1.393 (3)
O2—C8	1.424 (3)	C5—H5A	0.9300
O2—C9	1.426 (3)	C7—C8	1.495 (3)
O3—C11	1.412 (3)	C7—H7A	0.9700
O3—C10	1.425 (2)	C7—H7B	0.9700
O4—C16	1.209 (3)	C8—H8A	0.9700
O5—N3	1.241 (3)	C8—H8B	0.9700
O6—N3	1.227 (2)	C9—C10	1.500 (3)
N1—C15	1.366 (3)	C9—H9A	0.9700
N1—C13	1.367 (3)	C9—H9B	0.9700
N1—C12	1.469 (3)	C10—H10A	0.9700
N2—C15	1.312 (3)	C10—H10B	0.9700
N2—C14	1.361 (3)	C11—C12	1.529 (3)
N3—C15	1.429 (3)	C11—H11A	0.9700
C1—C2	1.374 (3)	C11—H11B	0.9700
C1—C6	1.393 (3)	C12—H12A	0.9700
C1—H1A	0.9300	C12—H12B	0.9700
C2—C3	1.400 (3)	C13—C14	1.375 (3)
C2—H2A	0.9300	C13—H13A	0.9300
C3—C4	1.396 (3)	C14—H14A	0.9300
C3—C16	1.467 (3)	C16—H16A	0.9300
C4—C5	1.388 (3)		
			
C6—O1—C7	118.52 (17)	C7—C8—H8B	109.8
C8—O2—C9	110.66 (17)	H8A—C8—H8B	108.3
C11—O3—C10	112.10 (16)	O2—C9—C10	109.2 (2)
C15—N1—C13	104.60 (19)	O2—C9—H9A	109.8
C15—N1—C12	130.7 (2)	C10—C9—H9A	109.8
C13—N1—C12	124.70 (19)	O2—C9—H9B	109.8
C15—N2—C14	104.12 (19)	C10—C9—H9B	109.8
O6—N3—O5	123.5 (2)	H9A—C9—H9B	108.3
O6—N3—C15	117.9 (2)	O3—C10—C9	108.74 (17)
O5—N3—C15	118.57 (18)	O3—C10—H10A	109.9
C2—C1—C6	120.3 (2)	C9—C10—H10A	109.9
C2—C1—H1A	119.8	O3—C10—H10B	109.9
C6—C1—H1A	119.8	C9—C10—H10B	109.9
C1—C2—C3	120.5 (2)	H10A—C10—H10B	108.3
C1—C2—H2A	119.7	O3—C11—C12	107.91 (18)
C3—C2—H2A	119.7	O3—C11—H11A	110.1
C4—C3—C2	118.5 (2)	C12—C11—H11A	110.1
C4—C3—C16	119.1 (2)	O3—C11—H11B	110.1
C2—C3—C16	122.4 (2)	C12—C11—H11B	110.1
C5—C4—C3	121.5 (2)	H11A—C11—H11B	108.4
C5—C4—H4A	119.2	N1—C12—C11	113.03 (19)
C3—C4—H4A	119.2	N1—C12—H12A	109.0
C4—C5—C6	118.7 (2)	C11—C12—H12A	109.0
C4—C5—H5A	120.6	N1—C12—H12B	109.0
C6—C5—H5A	120.6	C11—C12—H12B	109.0
O1—C6—C5	123.9 (2)	H12A—C12—H12B	107.8
O1—C6—C1	115.77 (19)	N1—C13—C14	106.8 (2)
C5—C6—C1	120.3 (2)	N1—C13—H13A	126.6
O1—C7—C8	107.08 (17)	C14—C13—H13A	126.6
O1—C7—H7A	110.3	N2—C14—C13	110.5 (2)
C8—C7—H7A	110.3	N2—C14—H14A	124.8
O1—C7—H7B	110.3	C13—C14—H14A	124.8
C8—C7—H7B	110.3	N2—C15—N1	114.0 (2)
H7A—C7—H7B	108.6	N2—C15—N3	122.39 (19)
O2—C8—C7	109.2 (2)	N1—C15—N3	123.6 (2)
O2—C8—H8A	109.8	O4—C16—C3	124.7 (2)
C7—C8—H8A	109.8	O4—C16—H16A	117.6
O2—C8—H8B	109.9	C3—C16—H16A	117.6
			
C6—C1—C2—C3	0.3 (3)	C15—N1—C12—C11	−69.6 (3)
C1—C2—C3—C4	0.3 (3)	C13—N1—C12—C11	108.9 (2)
C1—C2—C3—C16	−178.4 (2)	O3—C11—C12—N1	−105.5 (2)
C2—C3—C4—C5	0.1 (3)	C15—N1—C13—C14	0.0 (3)
C16—C3—C4—C5	178.9 (2)	C12—N1—C13—C14	−178.9 (2)
C3—C4—C5—C6	−1.2 (3)	C15—N2—C14—C13	−0.3 (3)
C7—O1—C6—C5	4.4 (3)	N1—C13—C14—N2	0.2 (3)
C7—O1—C6—C1	−175.6 (2)	C14—N2—C15—N1	0.3 (3)
C4—C5—C6—O1	−178.2 (2)	C14—N2—C15—N3	−177.8 (2)
C4—C5—C6—C1	1.9 (3)	C13—N1—C15—N2	−0.1 (3)
C2—C1—C6—O1	178.6 (2)	C12—N1—C15—N2	178.6 (2)
C2—C1—C6—C5	−1.4 (3)	C13—N1—C15—N3	177.9 (2)
C6—O1—C7—C8	179.05 (18)	C12—N1—C15—N3	−3.3 (4)
C9—O2—C8—C7	167.89 (17)	O6—N3—C15—N2	−5.4 (3)
O1—C7—C8—O2	−70.0 (2)	O5—N3—C15—N2	174.2 (2)
C8—O2—C9—C10	176.92 (17)	O6—N3—C15—N1	176.7 (2)
C11—O3—C10—C9	176.86 (19)	O5—N3—C15—N1	−3.7 (3)
O2—C9—C10—O3	−75.8 (2)	C4—C3—C16—O4	172.9 (2)
C10—O3—C11—C12	−171.84 (19)	C2—C3—C16—O4	−8.4 (4)

### Hydrogen-bond geometry (Å, °)

**Table:**

*D*—H···*A*	*D*—H	H···*A*	*D*···*A*	*D*—H···*A*
C9—H9B···O4^i^	0.97	2.56	3.335 (3)	137.
C10—H10A···O4^ii^	0.97	2.57	3.461 (3)	152.

Symmetry codes: (i) *x*+3/2, −*y*+3/2, −*z*; (ii) *x*+1/2, −*y*+3/2, −*z*.
